# Social engagement modulates wild monkeys’ vocal expressions and the behavioral response to that of others

**DOI:** 10.1016/j.isci.2025.114408

**Published:** 2025-12-11

**Authors:** Alice Galotti, Luca Pedruzzi, Martina Francesconi, Alberto Quartesan, Sheleme Abiyou Gamessa, Valentina Serra, Giulio Petroni, Bezawork Afework Bogale, Alban Lemasson, Elisabetta Palagi

**Affiliations:** 1 Department of Biology, University of Pisa, Via Alessandro Volta 6, Pisa 56126, Italy; 2 EthoS (Ethologie Animale et Humaine) - U.M.R 6552, Université de Rennes, Université de Normandie, CNRS, Rennes 35000, France; 3 Department of Zoological Sciences, College of Natural and Computational Sciences, Addis Ababa University, Addis Ababa 1000, Ethiopia; 4 Institut Universitaire de France, Paris 75231, France; 5 Natural History Museum, University of Pisa, Via Roma 79, Calci, Pisa 56011, Italy

**Keywords:** Wildlife behavior, Biological sciences, Zoology, Evolutionary biology

## Abstract

Animal vocal communication relies on the dynamic interaction between emitter and receiver, with signals shaped within a social and embodied context. To fully understand how such interactive processes operate, we used yawn vocalizations of geladas (*Theropithecus gelada*), a species showing exceptional yawning variability. We first examined yawn calls produced in three contexts: high-intensity social, low-intensity social, and non-social context and found clear acoustic differences among them, revealing context-dependent modulation in a typically stereotyped behavior. We conducted field playback experiments exposing wild geladas to unfamiliar male yawn vocalizations emitted in the three contexts. During playbacks, monkeys gazed more at the loudspeaker when yawns originated from a social rather than a non-social context, indicating that animals perceive the stimuli’s differing nature. Although yawn responses did not vary across contexts, contagion was higher when geladas were grooming during test, suggesting that positive social engagement enhances, rather than reduces, susceptibility to contagion.

## Highlights

• Gelada yawns vary acoustically across contexts, challenging their fixed nature
• Geladas show greater attention to yawns in social than in non-social contexts
• Yawn contagion occurs regardless of the emitter’s behavioral context
• Receiver context, such as grooming, increases susceptibility to yawn contagion

## Graphical abstract

**Figure:**
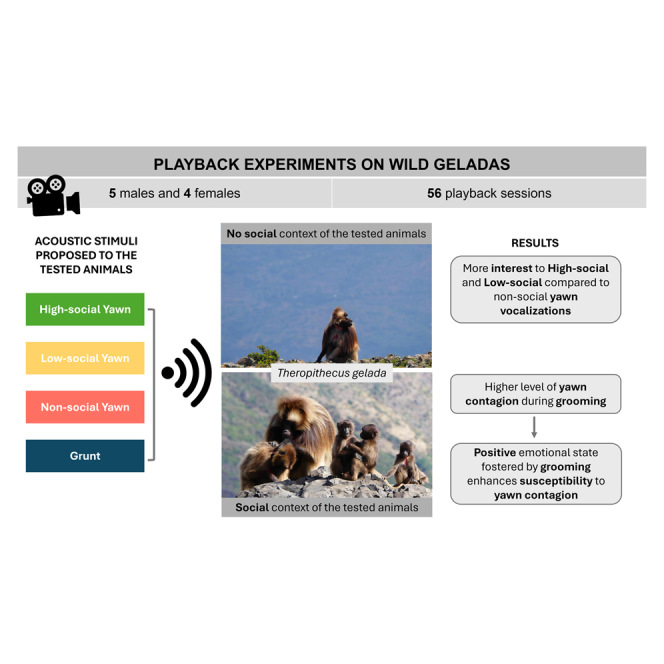

## Introduction

Vocal signals often evoke responses that are influenced not only by their acoustically encoded information but also by the context in which they are emitted and the internal state of the receiver.^1^^,^^2^ This context-dependent modulation of vocalizations allows individuals to convey different meanings or enhance the efficacy of signals in various social situations.^3^ As such, the capacity to increase the variability of information transmission in animal communication should be further explored, particularly as interpreting behavioral response is often not straightforward.^4^ While referential call types and other mono-contextual vocalizations are relatively rare, many call types are typically produced across a broad range of contexts. Many studies have highlighted context-specificity of vocalizations and their acoustic properties across various taxa, including birds,^5^^,^^6^ cetaceans,^7^^,^^8^ dogs,^9^ bats,^10^^,^^11^ and nonhuman primates.^12^^,^^13^ These findings highlight that changes in the acoustic structure of calls are linked to various behaviors, including food-related activities,^14^ agonistic interactions,^15^ and long-distance communication.^16^ Additionally, vocalizations encode emotional states, with specific call types linked to positive or negative valence (e.g., ultrasonic vocalizations in rats^17^), as well as acoustic features coding for low vs. high arousal.^18^ In addition to the fact that vocal parameters reflect the emitter emotional and social contexts,^19^ the same call may also elicit different responses depending on the receiver’s internal state, thus leading to the same call being perceived slightly differently. In meerkats (*Suricata suricatta*), subordinate females adjust their responses to dominant females’ vocalizations based on whether they had recently experienced a conflict or a neutral interaction, suggesting that responses are context dependent.^20^ Similarly, in baboons (*P*. *hamadryas ursinus*), females react more strongly to threat vocalizations from kin in stressful situations compared to non-kin or neutral situations.^21^ However, all the mentioned studies investigated contextual and/or affective changes to animal calls focusing on vocalizations that have primarily evolved for communicative reasons. Here, to avoid any referential confusing factor, we studied a state-based affected sound by focusing on the effect of the context on the expressive and perceptual components using as a model derived vocalizations shown by a non-human primate while yawning. It is a widespread behavior whose complexity and communicative functions are still highly debated.^22^^,^^23^^,^^24^^,^^25^ Despite its stereotypical nature, in humans yawning serves as a multimodal cue, in which the visual component is often integrated by vocalizations.^25^^,^^26^^,^^27^^,^^28^ The social significance of yawning is particularly evident in its contagiousness, triggering similar responses in others.^29^ The phenomenon of yawn contagion extends beyond our species, being observed in various non-human social species.^22^^,^^30^^,^^31^^,^^32^ Recent research further highlights the role of yawn contagion in synchronizing collective behavior in non-human social animals. In lions (*Panthera leo*), contagious yawning among group members facilitates behavioral alignment, ensuring coordinated activities.^33^ Such synchronization is essential for fostering social cohesion and supporting cooperative interactions within social species.^23^^,^^32^ Moreover, additional adaptive benefits of yawn contagion should be considered. In particular, some studies have explored its role in promoting group vigilance.^34^^,^^35^ From an evolutionary perspective, maintaining alertness to potential threats would provide an immediate survival advantage.

Geladas (*Theropithecus gelada*), a highly social monkey species endemic to the Ethiopian highlands,^36^^,^^37^ have a rich vocal repertoire^38^^,^^39^^,^^40^^,^^41^^,^^42^ and, along with humans,^28^ are the only primates known to produce specific derived vocalizations during yawning.^40^^,^^41^ Recent experimental and observational studies showed that the sound of a yawn is alone sufficient to elicit contagious yawning in the species,^41^^,^^43^ hinting at the communicative role of yawn vocalizations in the coordination of social interactions. However, data on the contextual and emotional triggers of yawn vocalizations, as well as the variability in their production and perception, are still lacking. Considering its role in group synchronization,^33^ the evolutionary advantage of auditory yawn contagion may lie in enabling individuals to maintain acoustic contact when visual information is prevented.^41^ This function could be particularly significant in societies characterized by modularity, flexibility, and high social complexity, where individuals rely on multimodal communicative strategies to manage interactions effectively.^40^^,^^44^^,^^45^^,^^46^ The high morphological variability of vocalized and non-vocalized yawns in geladas, ranging from yawns with covered teeth to those with uncovered teeth and gums,^47^ together with the wide range of contexts in which geladas yawn,^48^ suggest that yawn vocalizations may also exhibit a greater variability than expected, potentially serving different communicative functions. In geladas, the acoustic component of yawns is mainly produced by males.^41^

**Figure 1:**
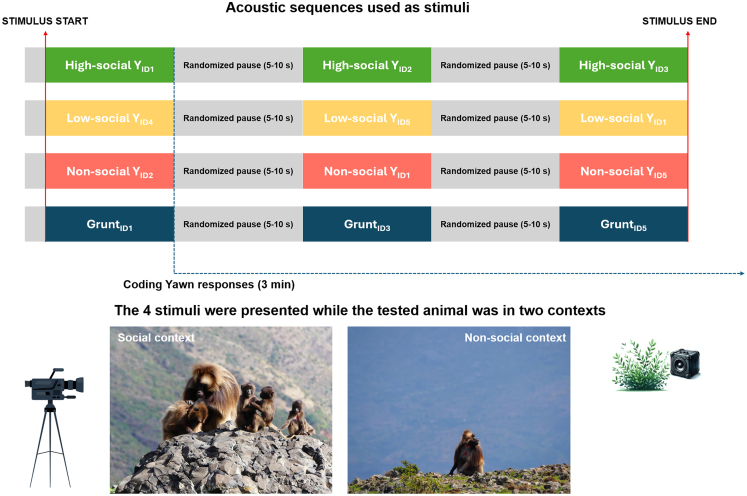
Acoustic stimuli and experimental contexts In the upper part, a graphical representation of the acoustic sequences used as stimuli is shown (green, high-social yawn vocalizations; yellow, low-social yawn vocalizations; red, non-social yawn vocalizations; and blue, grunt social vocalizations). “ID_number_” indicates the identity of the subjects that emitted the vocalizations (five captive gelada males). The blue dashed line indicates the moment when the coding of yawning responses began, which occurred immediately after the end of the first vocalization within the stimulus. Yawning responses were then recorded for the following 3 min. In the bottom part, two pictures illustrate the two contexts in which wild geladas were exposed to the playback experiments (social context vs. non-social context).

Here, we conducted field playback experiments on wild geladas, exposing the animals to unfamiliar male yawn vocalizations emitted during three different contexts: (i) high-social (e.g., post-mounting), (ii) low-social (when involved in grooming), and (iii) non-social (solitary activities) (See Figure 1).

We predicted that the acoustic structure of yawn vocalizations (expressive component *sensu* Tsutsui^49^) would vary depending on the context in which they are emitted (*Prediction 1*) and that geladas are able to distinguish the contextual origin of different yawn vocalizations (perception component *sensu* Mateo^50^), differently responding to yawns recorded during a social context compared to yawns recorded in a non-social context (i.e., increased number of gazes directed to the loudspeaker) (*Prediction 2*). Furthermore, we hypothesized an increase of yawn contagion when the context of the stimulus broadcasted has a social compared to non-social origin (*Prediction 3a*). If the socio-emotional context experienced by the tested subjects during the stimulus administration has a role in modulating the yawn contagion response,^20^^,^^21^^,^^51^ two alternative scenarios are possible. If an individual is engaged in grooming, its focus on the ongoing interaction might reduce the motivation to respond to external stimuli (*Prediction 3b*); if the social engagement produced by grooming fosters the receiver’s positive emotional state, we expect a higher motivation to respond to yawn vocalizations during grooming than during a non-social situation (*Prediction 3c*).

## Results

### Acoustic analyses

**Figure 2:**
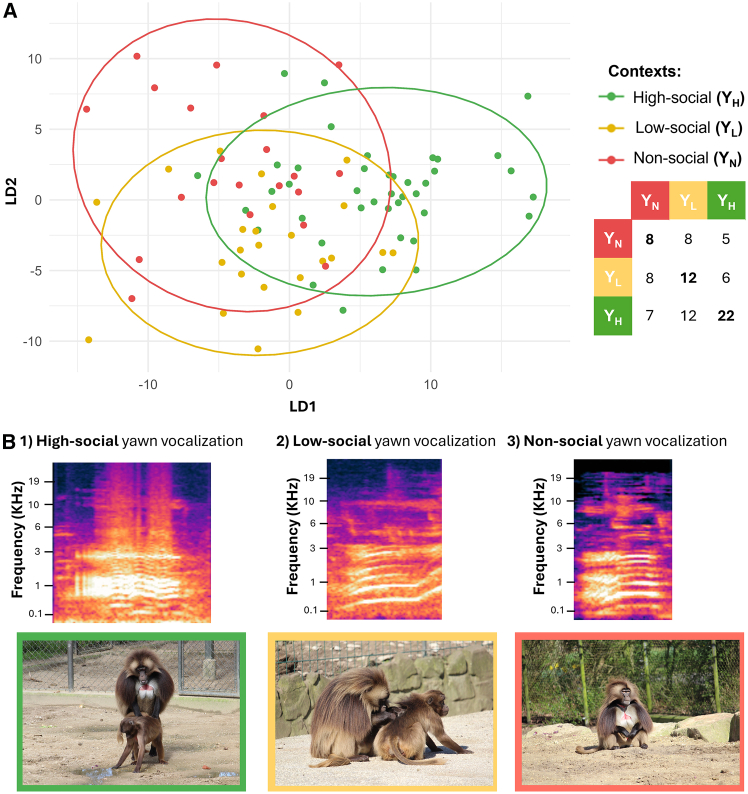
Acoustic classification and spectral structure of yawn types (A) The DFA plot represents the classification of yawn vocalizations based on their acoustic features. The data points are color-coded according to the three different contexts in which yawn vocalizations were recorded: green for high-social (Y_H_), yellow for low-social (Y_L_) and red for non-social yawn vocalizations (Y_N_). The axes correspond to the two discriminant functions (LD1 and LD2), which maximize the separation between the categories based on their acoustic properties. Each dot represents a single vocalization, while the ellipses indicate the confidence intervals around the group centroids, showing the overall distribution of each vocalization context. The classification matrix (right panel) displays the proportion of correctly classified vocalizations for each category. (B) Spectrograms of yawn vocalizations for the three different contexts: (1) high-social, (2) low-social, and (3) non-social. Pictures representing each of the three contexts are also included.

Using the selected 18 parameters as independent variables, the discriminant function analysis (DFA) correctly classified the context of yawn vocalization with an accuracy of 48% (Figure 2), which was statistically above chance (binomial test, *p* = 0.006, Table 1). Specifically, the accuracy of the DFA classification for each context was 38% (binomial test, *p* = 0.129) for non-social yawn vocalizations, 46% for low-social yawn vocalizations (binomial test, *p* = 0.08), and 54% for high-social yawn vocalizations (binomial test, *p* = 0.434). In the Figure S2 we reported the influence of each acoustic features to the first two discriminant functions. See Figure 2B for spectrograms illustrating each context.

**Table 1:** Acoustic classification of gelada yawns across contexts

Yawn contexts	Chance probabilities	DFA correct assignment proportion	Binomial test (*p* value)
High-social	0.238	0.381	0.129
Low-social	0.295	0.461	0.831
Non-social	0.465	0.537	0.434
Total correct classification after cross-validation	0.333	0.477	**0.006**

Results of DFA on the 18 acoustic parameters of gelada yawns from three different contexts (high-social, low-social, non-social). Statistically significant results are highlighted in bold.

For each playback session we reported in the Table S2 (Model 1, 2, and 3) and Table S3 (Model 4) the number of gazes toward the loudspeaker, the number of self-directed behaviors and yawn presence/absence both before and after the stimulus administration.

#### Model 1

**Figure 3:**
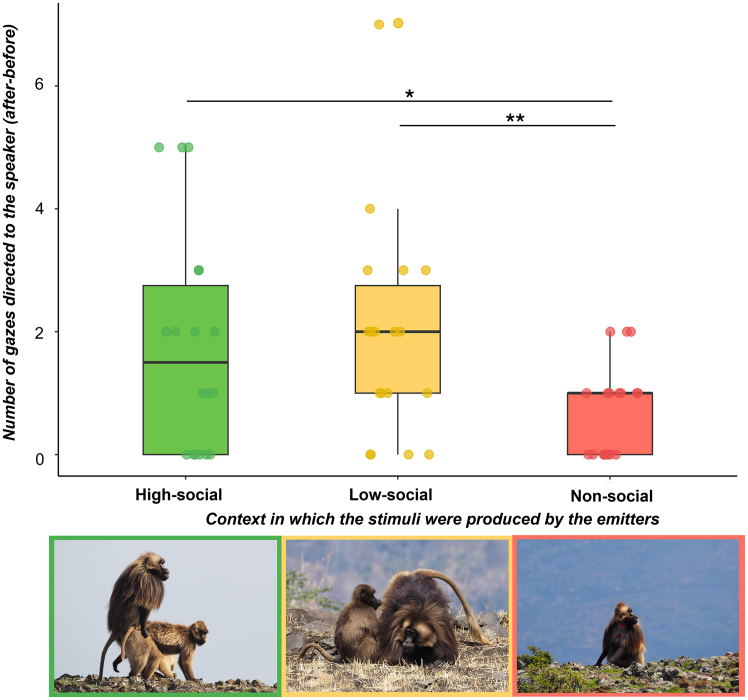
Gaze responses to playback stimuli across contexts The boxplot represents the number of gazes (after, before the stimuli) at the loudspeaker under high-social (green), low-social (yellow), and non-social (red) contexts. Asterisks indicate statistical significance, *p* < 0.05 (∗), *p* < 0.01 (∗∗), based on GLMM. The dots indicate raw data. The boxes display the median value and the first and third quartiles, while whiskers extend to the most extreme values within 1.5 times the interquartile range (IQR).

The *number of gazes to the speaker* (the difference between the number of gazes after and before the stimulus perception) was the response variable. The full model was significantly different from the control one (χ^2^_4_ = 14.967, *p* = 0.004; Table 2). The results show that the number of gazes directed to the loudspeaker was higher when the yawn vocalization was produced in a social (either high-social or low-social) compared to a non-social context (t ratio_highsocial-lowsocial_ = −0.617, *p* = 0.853, t ratio_highsocial-nosocial_ = 2.602, *p* = 0.024, t ratio_lowsocial-nosocial_ = 3.314, *p* = 0.002, Figure 3 and Table S2).

**Table 2:** Model estimates and likelihood ratio tests

Fixed effects	Coeff	SE	χ2	df	P
**Model 1 (GLMM) – the number of gazes to the speaker**					

Intercept	1.192	0.349	N/A	N/A	N/A

**Tested variable**					

Sex	0.331	0.284	1.355	1	0.244
Context_receiver_	−0.095	0.249	0.146	1	0.702
Context_trigger_	–	–	11.448	2	**0.003**
Context_triggerL_	0.162	0.301	–	–	–
Context_triggerN_	−0.938	0.358	–	–	–

**Control variables**					

**Trial order**	−0.184	0.056	10.694	1	**0.001**
Variance for the random factors: ID_subject_ = 0.03519 ± 0.1876SD; N_observation_ = 54; N_subjects_ = 9.					

**Model 3 (GLMM) – presence/absence of yawn responses**					

Intercept	−1.786	1.656	N/A	N/A	N/A

**Tested variables**					

Sex	−1.799	1.080	2.775	1	0.096
Context_receiver_	3.162	1.290	6.008	1	**0.014**
Context_trigger_	–	–	4.175	2	0.124
Context_triggerL_	−1.861	1.122	–	–	–
Context_triggerN_	−2.042	1.160	–	–	–

**Control variables**					

Trial order	−0.184	0.056	0.033	1	0.856
Number of gazes directed to the speaker	0.736	0.604	0.007	1	0.929
Variance for the random factors: ID_receiver_ = 0.001 ± 0.001SD; N_observation_ = 54; N_subjects_ = 9.					

**Model 4 (GLMM) – responses to grunt or yawn vocalizations**					

Intercept	6.785	11.520	N/A	N/A	N/A

**Tested variables**					

Context_trigger_	51.396	23.495	4.785	1	**0.028**

**Control variables**					

Sex	−38.114	18.767	4.124	1	**0.042**
Trial order	−6.583	3.761	3.064	1	0.080
Variance for the random factors: ID_receiver_ = 21828 ± 147.7SD; N_observation_ = 18; N_subjects_ = 9.					

Estimated parameters (Coeff), standard error (SE), and results of the likelihood ratio tests (**χ**^**2**^) of the *MODEL*_*response*_, *MODEL*_*occurrence*_ and *MODEL*_*latency*_. Significant *p* values are shown in bold; df = degree of freedom; n/a = not applicable.

#### Model 2

The *number of self-directed behaviors* (the difference between the number of bouts of self-directed behaviors after and before the stimulus perception) was the response variable. The full model did not significantly differ from the control one (χ^2^_4_ = 2.630, *p* = 0.621, Table S2).

#### Model 3

**Figure 4:**
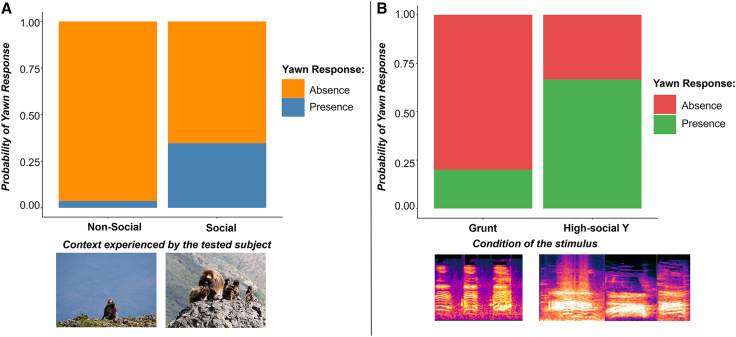
Probability of yawning responses across experimental conditions (A) The bar chart represents the probability of yawning responses (orange, absence and blue, presence) in the social and non-social contexts experienced by the receiver. (B) The bar chart shows the probability of yawning responses (green, presence and red, absence) following exposure to a grunt and yawn vocalizations (Y) both recorded under social context. In the bottom part, two spectrograms illustrating the acoustic structure of three grunt and three yawn vocalizations.

The *presence/absence yawn responses* by the tested animal was the response variable. The full model was significantly different from the control one (χ^2^_4_ = 16.562, *p* = 0.002). The results show that the probability for the tested subject to respond with a yawn to the stimulus was higher when he/she was involved in grooming as *context* during the experiment than during non-social activities (feeding/resting) (Tables 2 and S2, Figure 4A), independently from the type of vocal stimulus administered.

#### Model 4

The *presence/absence yawn responses* was the response variable. The full model was significantly different from the control one (χ^2^_1_ = 11.379, *p* < 0.01; Table 2). The results show that the probability to obtain a yawn response in playback experiments during grooming was higher after perceiving a high-social yawn acoustic stimulus compared to a grunt (thus emitted in a comparably social context). Descriptive statistics show that the probability of a yawn response following high-social yawn playbacks was 55.6%, whereas responses following grunt playbacks were observed in 22.2% of trials (Table S3). This indicates that, although after grunts occasionally we recorded yawns, the response rate remained substantially lower than for high-social yawns (Figure 4B).

## Discussion

Our results provide valuable insights into the relationship between the sender’s social contexts at the time of vocalization emission and call acoustic structure, as well as the relationship between the social context experienced by the receiver and their behavioral response to others’ vocalizations. Our study investigated a derived vocalization, the *yawn call*, which uniquely emerged in humans and gelada monkeys and is produced across a range of contexts. First, the DFA revealed systematic variations in the acoustic parameters of male yawn vocalizations produced across different social contexts with classification accuracy significantly above chance, confirming a non-random acoustic variability (Prediction 1 supported). High-social (produced after mounting, an affiliative behavior in geladas,^52^ or vocal interactions, affiliative derived calls with females^52^), low-social (during grooming), and non-social yawns (solitary activities) were possibly associated with different degrees of emotional arousal. This suggests that variations in yawn vocalizations may unconsciously encode contextual information, indicating a wider within-call contextual variability allowing to increase the range of information conveyed, as it occurs for other primate vocalizations.^13^ Our results are also in line with previous findings on other gelada vocalizations, grunts, which were found to vary according to the behavioral context in which they are produced.^53^ This unexpected finding calls into question the very characterization of yawning as a fixed action pattern,^29^ revealing a level of flexibility that contradicts its traditionally assumed rigidity, not only in its visual,^54^^,^^55^ but also in its acoustic component. Although the overall classification accuracy obtained through the DFA was moderate (48%), this outcome is not unexpected given that our analysis focused exclusively on the contextual dimension and did not include other potentially relevant sources of variation (e.g., individual identity, age, body size, or yawn morphology). Moreover, probably yawn calls are not signals selected to convey specific contextual meanings possibly explaining the partial overlap among categories. The differences found raise interesting questions about the meaning and underlying mechanisms of such inter-contextual variability. They may indicate that yawning-associated vocalizations convey different information depending on context, for instance, during grooming (e.g., a relaxed state) versus after sexual interactions or intense cross-sex greetings (e.g., high-arousal or other cues involuntarily expressed toward neighboring males). Playback experiments showed that geladas gazed more frequently at the loudspeaker when exposed to vocalizations deriving from yawns emitted in both high- and low-social context compared to a non-social context. Furthermore, the absence of a significant difference in gazing at high- and low-social stimuli suggests that receivers are sensitive and/or motivated to react to yawn vocalizations whatever the level of positive social arousal the yawner is experiencing. This finding provides evidence for the perceptual component of the cue,^50^ indicating that yawn vocalizations convey to some extent information about the social aspect of the context of production, thereby supporting Prediction 2. The ability to distinguish between socially relevant and less relevant yawns suggests an adaptive benefit in modulating attention to group-related cues, particularly in species with complex social dynamics.^56^

While the interest toward the stimuli is influenced by their social or non-social origin, the same does not apply to yawn responses, as yawn contagion was consistently elicited by the broadcasted yawns regardless of the context (high-social, low-social, or non-social) in which they were recorded (Prediction 3a not supported). This finding aligns with existing knowledge on yawn contagion across species, including humans. In *Homo sapiens*, it is well established that merely hearing or reading the word “yawn,” or even thinking about the behavior in the absence of any specific context, can automatically and unconsciously trigger the same behavioral pattern in many cases.^29^ The automaticity of yawn contagion is shown in geladas, where the responses do not seem to vary according to the modality of perception, the morphology of the yawn or the side of perception.^43^ The absence of a direct link between the stimulus contextual origin and yawn contagion further underscores the need to consider not only the external (e.g., emission context) factors but also the internal states of the receivers (e.g., social engagement at the time of exposure, internal affective state) in shaping behavioral responses. Our data support this last consideration. Specifically, geladas exhibited higher levels of yawn contagion in response to the stimuli while engaged in grooming compared to when involved in solitary activities, albeit their interest for the loudspeaker (i.e., gazing) was not affected by such activities. Yet, this finding is not sufficient *per se* to confirm that an ongoing grooming interaction directly facilitates yawn contagion. To further explore this hypothesis, we needed to exclude the possibility that the increased yawn contagion during grooming was not simply due to an increase in spontaneous yawning but actually due to an increased propensity to respond to others’ yawn vocalizations. To exclude this possibility, we administered to geladas engaging in grooming activities yawn and grunt vocalizations both recorded during the same social context. The sound of social yawns evoked a higher yawn response than social grunts. This suggests not only that being actively involved in a social interaction does not reduce contagion susceptibility to external vocal yawns, but that the positive emotional state deriving from grooming might foster such susceptibility. To aid interpretation of the playback results, it is essential to consider the naturally high spontaneous yawning rates in geladas. A previous study showed that gelada adult males yawn up to 0.25 times per minute,^57^ making the probability of at least one spontaneous yawn within 3 min exceed 20%–25%. This may explain the presence of yawn responses after grunts playbacks. Moreover, our last analyses included only grooming sessions, a context in which spontaneous yawning is very common in geladas.^57^ Consistent with this, yawning responses during grooming sessions occurred across all stimuli conditions. Nevertheless, the probability of responding to high-social yawn calls remains substantially higher, supporting the interpretation that socially relevant (and emotionally aroused) yawning calls elicit a stronger response than other types of social or non-social vocalizations.

Our study was conducted in fully natural settings, where logistical constraints naturally limit sample size. However, the number of subjects in our study is consistent with standards in playback research across nonhuman primates and other mammals (chimpanzees,^58^ bonobos,^59^ geladas,^60^ and cheetahs^61^). Combined with repeated, controlled exposures per individual and careful spacing of sessions, this design ensures robust within-subject comparisons and meaningful insights into context-dependent communication and yawn contagion.

In conclusion, yawn vocalizations emitted both in high- and low-social contexts exhibit structural differences compared to those produced in non-social contexts, reflecting the expressive component of the vocal cue. This unexpected finding challenges the assumption that yawning is a purely stereotypic behavior, suggesting instead that it may carry nuanced social and communicative functions. Moreover, geladas seem to perceive them as distinct, showing a stronger interest (measured via their gazing behavior) to yawn calls emitted during social interactions, highlighting the perceptual component of the vocal cue. However, yawn contagion does not appear to depend on the context in which the cue originates but rather on the context currently experienced by the receiver. Specifically, positive contexts increase the likelihood of a contagious response. This “*hic et nunc*” effect, evidenced by our study, calls for a critical reconsideration of correlational data suggesting that yawn contagion covariates, both positively or negatively, with specific social variables such as relationship quality,^47^^,^^62^^,^^63^^,^^64^ kinship,^31^^,^^65^^,^^66^ or social rank.^30^^,^^67^ If the immediate context modulates the yawn response, then correlational findings may not accurately reflect the actual dynamics of yawn contagion.^33^ For instance, if an individual is engaged in a grooming session with a closely bonded partner and perceives a yawn from another, less socially connected individual, the likelihood of a contagious response may be more influenced by the ongoing grooming activity than by the relationship with the yawning individual. A grooming session represents a kaleidoscope of underlying factors, including focused attention on conspecifics, ectoparasite removal, physical stillness, and a distinctive somatosensory experience, not to mention a potential reduction in alertness to external threats. Consequently, it is challenging to disentangle which of these elements may actually influence yawn contagion. What does seem evident, however, is that the activity being performed by the individual at the time of exposure substantially modulates their response.

Taken together, our results further highlight the remarkable socio-communicative complexity of geladas, which is reflected even in seemingly simple behaviors such as yawning and yawn-related vocalizations. Moreover, our findings raise intriguing questions about the potential communicative complexity of yawn calls in our own species. From this perspective, if we truly aim to unravel the proximate and ultimate drivers^68^ of yawn contagion, our attention must shift toward the immediate effects and underlying mechanisms that shape this phenomenon in real time. Only by embracing a dynamic, context-dependent approach can we move beyond mere correlations and capture the true nature of this intriguing social behavior. This shift in perspective not only refines our understanding of yawning and yawn contagion but also paves the way for deeper insights into the complex interplay between social context, perception, cognition, and behavioral synchronization.

### Limitations of the study

Our study provides novel insights into the acoustic and social dynamics of yawn vocalizations in geladas; however, some limitations should be acknowledged. First, the sample size, although sufficient to detect systematic acoustic differences and contagion patterns, and comparable to those used in similar studies, was relatively small. A larger dataset would have allowed us to explore additional contexts, include more individuals, and increase the number of yawns per context. Second, although our observations were conducted under naturalistic conditions, we did not directly assess internal factors such as individual temperament or stress levels, which may influence yawning behavior. Third, we used stimuli collected from a different (captive) population to minimize potential biases related to familiarity; nonetheless, we cannot entirely rule out perceptual differences arising from inter-population variation. Finally, our analyses focused primarily on male vocalizations, as they are more frequent and conspicuous than those of females, leaving potential acoustic differences in female yawns for future investigation.

## Resource availability

### Lead contact

Further information and requests for resources and reagents should be directed to and will be fulfilled by the lead contact, Elisabetta Palagi (elisabetta.palagi@unipi.it).

### Materials availability

No new materials were generated.

### Data and code availability

• Raw audio and video recordings, as well as processed acoustic measurements, are available from the corresponding author upon request.
• Scripts used for audio analysis and statistical modeling are available from the corresponding author upon request.
• Hardware specifications, recording equipment, and software versions are detailed in the key resources table; no additional materials are required.

## Supplemental information

Document S1. Figures S1 and S2, Tables S1–S3

Data S1. Dataset for the first three models

Data S2. Dataset for the last model

## Acknowledgments

We would like to express our sincere gratitude to the Ethiopian Wildlife Conservation Authority (EWCA) and the Oromia Forest and Wildlife Service for granting us permission to conduct our research. Our heartfelt thanks also go to the Debre Libanos Woreda and Monastery for their generous support during our stay in the community. We are deeply grateful to Achim Johann, the coordinator of the Gelada European Endangered Species Programme, for his invaluable assistance in fundraising. A special thank you to Birhanu and, above all, Kabebew Asefa Ylma for their unwavering care and support in the field. We sincerely thank Hélène Bouchet for her valuable advice on the acoustic analyses. Finally, we wish to thank every single kid of Debre Libanos for gifting us a smile every day during our stay.

The research has been funded by the Leakey Foundation (Science for reconciliation: What an Ethiopian monkey tells about peace-making, grant n° S202310431, PI Elisabetta Palagi) and by the following zoos and foundations (funders of BRIDGES project, UNIPI, AOO “BIO” – 0005878/2022, PI Elisabetta Palagi), in alphabetic order: Dudley Zoo (UK), Fondazione ARCA (Italy), Giardino Zoologico di Pistoia (Italy), NaturZoo Rheine (Germany), Parc des Félins (France), Parco Natura Viva (Italy), Parco Zoo Falconara (Italy), Rotterdam Zoo (The Netherlands), Saint-Félicien (Canada), Wildlife Conservation Benefit (Italy), Wilhelma Zoo (Germany), Zoo de Cerza (France), Zoo de La Boissière du Doré (France). Rennes Métropole and the Région Bretagne (France) covered the field expenses for the first author while the University of Pisa and University of Rennes funded student fellowships.

## Author contributions

Conceptualization, A.G., L.P., E.P., and A.L.; data collection, A.G., L.P., M.F., and A.Q.; video analyses, A.G. and A.Q.; statistical and acoustic analyses, A.G. and L.P.; investigation, A.G., L.P., M.F., and A.Q.; visualization, A.G.; supervision, A.L. and E.P.; writing – original draft, A.G. and E.P.; writing – review & editing, A.G., L.P., M.F., A.Q., S.A.G., V.S., G.P., B.A.B., A.L., and E.P.

## Declaration of interests

The authors declare no competing interests.

## STAR★Methods

### Key resources table

**Table:** 

REAGENT or RESOURCE	SOURCE	IDENTIFIER
**Biological samples**		

Geladas (male yawners)	NaturZoo Rheine, Germany	Five adult males, individually identified
Wild geladas (playback subjects)	Debre Libanos, Ethiopia	Nine adult subjects (5 males, 4 females)

**Software and algorithms**		

Audio editing software	Audacity 3.3.2	RRID: SCR_007198
Statistical software	R Studio	Version 4.x; RRID: SCR_000432

**Other**		

Microphone	Sennheiser MKE600	–
Recorder	ZOOM H5	WAV, 44.1 kHz, 16-bit
Speaker	MiPRO MA-100 Personal Wireless PA	–
Video camera	SONY Handycam FDR-AX43A	–

### Experimental model and study participant details

Playback experiments were conducted from October to December 2024 in the unprotected area of Debre Libanos (North-West Shewa zone, Oromia regional state, Ethiopia, altitude 2150–2650 m^69^). The study site, a grazing area locally called Godot, provided optimal conditions for playback experiments (e.g., good visibility of the animals, natural objects to hide the loudspeaker and no barriers to sound propagation) (Figure S1). For six days a week from 08:00 a.m. to 14:00 p.m., seven one-male units (OMUs) regularly visiting the study site were followed. The initial ten days served to familiarize geladas to the presence of cameras and operators and to spread awareness about the research in the local communities, as geladas are often considered pests.^70^ During this interval, OMUs were identified based on the dominant males’ body features (e.g., size, fur color, scars, facial traits, and individual marks) and group size and composition. All adult females were recognized based on chest features (e.g., shape of nipples, number and position of vesicles). The tested subjects included five fully grown males and four adult females, all individually recognized and accustomed to human presence, allowing researchers to observe without causing any form of disturbance.

### Method details

#### Ethic statement

Although the study was entirely non-invasive, formal approval was obtained from the Bioethical Committee of the University of Pisa (OPBA, n. 14/2023). All research procedures complied with the laws and approved guidelines of the Ethiopian Wildlife Conservation Authority (EWCA). The study involved wild adult geladas of both sexes.

#### Acoustic recordings and selection of vocalizations

In 2023 (April-May), LP and MF collected various yawn and grunt vocalizations produced by gelada males at the NaturZoo Rheine in Germany. We selected vocalizations of males because more conspicuous than those of females.^41^^,^^43^ These vocalizations were recorded using a directional microphone (Sennheiser© MKE600) linked to a handy recorder (ZOOM H5©, sample rate: 44,100 Hz, resolution: 16-bit, wav format) at a distance of 5–15 m. The five adult leader males that emitted the vocalizations were individually identified by the two operators and were completely unknown to the wild subjects. We selected three contexts in which yawn vocalizations are generally produced: (i) high-social context - immediately after male-female mounting, a social behavior employed during affiliative interactions in geladas^52^ or male-female greetings during which the male approach the female producing sequences of vocalizations^71^; (ii) low-social context - grooming; and (iii) non-social context - solitary activities such as resting or feeding. Importantly, the two social contexts we classified probably differ not just in their level of social complexity but also in their intrinsic nature. While mounting in geladas may also serve affiliative purposes (Swedell, 1997), our categorization was based mainly on the expected differences in intensity and arousal between the two interactions. Moreover, we also recorded grunts, affiliative vocalizations commonly emitted in social contexts.^71^ During the recording phase, loudness (in decibels, dB) of each vocalization was measured using a professional sound meter (SLM-25, Gain Express Holdings©). Only recordings with a high signal-to-noise ratio were used, excluding those with overlapping sounds (e.g., bird calls or vocalizations from other geladas). After the stimulus collection, recordings were edited using Audacity© software (version 3.3.2) to create a pool of yawns and grunts for constructing the final stimuli.

#### Acoustic analysis

To assess if yawns emitted in the three contexts (high-social, low-social and non-social contexts) acoustically differed, a discriminant function analysis (DFA) was used; this is a supervised analysis that uses extracted parameters to maximize differences between the vocalizations of each context. For each vocalization, three R packages (*seewave*, *tuner*, *soundgen*) were used to extract a series (*n* = 178) of spectral and temporal acoustic parameters, which were potentially important to discriminate between contexts. These included both temporal measures, such as vocalization duration, and intensity measures, related to lung capacity, source-related vocal features (*f*0), and filter-related acoustic vocal features (formants).^72^^,^^73^^,^^74^^,^^75^ For each acoustic parameter an index adjusted from the Potential of Individual Coding (PIC) was calculated, the Potential of Contextual Coding (PCC). To control for individual acoustic variation, we balanced the dataset by selecting a nearly equal number of vocalizations for each of the five male geladas across the three different contexts (e.g., the same subject vocalization never used more than twice). This approach minimized individual overrepresentation in any specific context, reducing potential biases in the PCC and DFA analyses. The PIC originally assesses the ratio between within-individual variation (CVw) and between-individual variation (CVb) of an acoustic parameter (which in our case were calculated considering the context and not the individual as grouping categories) using the formula: $\frac{CVb}{mean\;CVw}$. The mean CVw is the mean value of the CVw for all contexts.^76^ The CVw calculated was the corrected formula for small samples (e.g., see^77^): $CVw=100\;\left(\frac{\text{standard}\;\text{deviation}}{X\;\text{mean}}\right)\left(1+\frac{1}{4n}\right)$, where *X* mean was the mean of the sample, and *n* was the sample size for one context. The CVb was calculated according to the formula: $CVb=100\;\left(\frac{\text{standard}\;\text{deviation}}{X\;\text{mean}}\right)$, where the standard deviation and *X*mean were calculated for the total sample. According to several studies,^72^^,^^73^^,^^74^^,^^78^^,^^79^ acoustic parameters with PIC ≥1 have potential to encode individual identity information, since their intra-individual variability is smaller than their inter-individual variability. Similarly, acoustic parameters with PCC ≥1 (Table S1) were used for further analyses. Indeed, to assess whether calls emitted in the three contexts differed acoustically, Discriminant Function Analysis (DFA) was used. Yawn vocalizations emitted in the three contexts were compared using a Discriminant Function Analysis (DFA) based on a final set of 18 acoustic parameters. These were identified through a three-step selection process: (i) only parameters showing a PCC greater than 1 were retained, (ii) parameters that did not meet the assumption of normal distribution were excluded, and (iii) to avoid redundancy and multicollinearity among highly correlated descriptors (e.g., mean, median, and standard deviation of the same feature), only one representative measure, typically the median, was kept for each set. The normality of the final parameters was further confirmed through visual inspection and Shapiro-Wilk tests.^80^ Following previous research,^73^^,^^78^^,^^79^ a leave-one-out cross validation was used (LOOCV) to assess the results of the DFA. LOOCV takes one vocalization out of the sample, runs the DFA with all other vocalizations, and then attempts to assign the excluded vocalization to the correct context. One-tailed binomial tests (*binom*.*test* function in *stats* library from *R Studio*) were used to detect significant differences in DFA with LOOCV for the whole set of calls and for each of the three contexts (high-social, low-social and non-social).

#### Analysis of variable contribution to discriminant functions

To determine how much each acoustic feature contributes to the discriminant functions derived from the DFA, the correlation between the original variables and the positions of the observations in the new discriminant space were evaluated. However, since the analysis employed leave-one-out cross-validation (CV = TRUE), the coefficients for the discriminant functions (scaling) were not available. Instead, the significance of each variable was approximated by measuring its correlation with the discriminant function scores (pred$x). To understand these relationships, a correlation matrix was calculated using R’s cor() function, revealing how each acoustic characteristic aligned with the discriminant axes. Higher correlation values (in absolute terms) implied a stronger impact of specific features on distinguishing between contexts along the discriminant dimensions. These correlations were further illustrated by creating a heatmap with the ggplot2 package. The correlation matrix was reshaped into a long-format table using the melt() function from the reshape2 package, making it suitable for visual representation. The heatmap used a color scale from blue (negative correlations) to red (positive correlations), with white representing negligible correlations. This approach facilitated evaluating the importance of variables in DFA, especially when direct access to the discriminant function coefficients was limited by cross-validation.

#### Stimulus preparation for the playback experiment

Four types of stimuli were composed, each including three consecutive calls emitted by the zoo-housed gelada males^41^: (i) control stimulus, three grunts performed by three males; (ii) high-social context, three yawn vocalizations from three males; (iii) low-social context, three yawn vocalizations from three males; and (iv) non-social context, three yawn vocalizations from three males (Figure 1). All vocalizations in each stimulus (yawns/grunts) were separated by a randomized pause of 5–10 s, obtaining stimulus from a minimum to 14 s to a maximum of 23 s. To prevent bias from loudness variation, the amplitude of all vocalizations was normalized. Before starting experiments and outside the experimental area, speaker volume was calibrated to approximately 60.0 dB.^41^^,^^61^^,^^81^ The pool of recordings used for stimuli creation included 30 grunts, 41 high-social yawn vocalizations, 26 low-social yawn vocalizations and 21 non-social yawn vocalizations. Each of the five captive males contributed equally to the dataset to prevent pseudo-replication. The four stimuli were randomly proposed to the nine study subjects when they were involved in two different contexts: social context (i.e., grooming) and non-social context (i.e., feeding or resting alone). At the end of the field data collection, each tested subject underwent a total of eight playback sessions (Figure 1). Each yawn or grunt vocalization was used no more than twice, never administered to the same subject and never in the same combination with other vocalization of the stimuli.

#### Experimental procedure

To minimize habituation and control for confounding factors, several precautions were implemented.^41^^,^^82^^,^^83^^,^^84^ Environmental conditions and the geladas tolerance enabled us to maintain a consistent distance (∼10 m) between the speaker and the animals during all playback sessions. To avoid simulating the vocal presence of an unfamiliar male within a familiar OMU, no gelada was present behind the speaker (MiPRO© MA-100 single-channel Personal Wireless PA system). No aggression should occur within 30 min prior to each playback, and no yawns should be visually or acoustically detected by researchers during the 3 min preceding stimulus administration. This exclusion criterion ensures that any yawns recorded during the playback can be confidently attributed to the stimulus itself, rather than to yawns performed by other group members in the immediately preceding period. During the experiments, one observer (AG, LP, MF, AQ) remotely activated the stimulus (in.wav format) via Bluetooth connected to a speaker hidden in the vegetation. Another observer (AG, LP, MF, AQ), generally visible to the focal gelada, video-recorded the subject (SONY© Handycam Full HD FDR-AX43A) for 1 min before and 3 min after the stimulus was broadcast. To ensure no prior yawns influenced the trial, a minimum waiting period of 3 min was observed after setting up the speaker. The video recorder’s direction was oriented at least 90° away from the direction of the acoustic stimulus. Due to the multi-level social structure of the species, other one-male units (OMUs) were always in proximity within ∼50 m of the focal individual. The subject had to remain visible, and no events likely to alter vigilance (e.g., arrival of new groups, aggressive interactions, or vocalizations from group members) should occur during playbacks. No more than three playbacks were conducted per day, and the same context stimulus was never repeated on the same day.

To prevent repeated exposure within short intervals, the identities of non-focal subjects, potentially hearing the stimuli, were recorded to ensure a minimum of 24 h between any two instances involving the same animal (no subject heard two stimuli in less than 24 h; in 12 cases the interval was between 24 and 48 h, and in the remaining 60 cases it exceeded 48 h**)**. This interval was considered appropriate to prevent potential habituation effects.^84^ Yawn calls naturally occur at relatively high frequencies within gelada groups,^43^ and, compared to other vocal stimuli, are less likely to elicit habituation in their contagious responses, as yawn contagion is an automatic, involuntary phenomenon that is difficult to suppress. Importantly, the order of playback sessions was included as a covariate in the statistical models to account for any systematic influence of repeated exposure on subjects’ responses. Once a subject was selected to undergo the playback experiment, the condition of the stimulus was randomly chosen. In the end, each subject received all four stimuli in the two contexts (social and solitary), with no stimulus presented more than once in the same context to the same individual. Therefore, although the order of presentation was random, the design ensured that each stimulus was equally represented across contexts, avoiding potential bias (Figure 1). Additionally, mock trials were conducted to accustom the geladas to the equipment and researchers, replicating the experimental setup without broadcasting any stimulus. Study groups were followed and recorded daily throughout the experimental period.

#### Video coding

Video recordings were analyzed frame-by-frame with the program PotPlayer (accuracy 0.02 s), coding the following behaviors: (i) number of gazes directed to the speaker in the minute before and after the stimuli; (ii) total looking time to the speaker in the minute before and after the stimuli; (iii) number of self-directed behaviors bout in the minute before and after the stimuli (scratching, self-grooming, proxy for anxiety state in primates^85^; (iv) total time of self-directed behaviors to the speaker in the minute before and after the stimuli; and (v) presence or absence of yawns in the 3 min following the start of the stimuli.^41^ Every instance in which the tested subjects turned their head toward the direction of the speaker was coded as looking at it. In primates, measures of the time subjects change head orientation after playback are commonly used to evaluate their general interest in a stimulus.^58^^,^^82^ In the analyses, the number of gazing events directed at the loudspeaker was calculated as the difference between this variable after and before the stimulus for each condition proposed to each tested subject; the same approach was applied to the number of self-directed behavior bouts. Behaviors were coded also before stimulus onset to measure the increase/decrease of interest toward the speaker area after the stimulus compared to before.^86^^,^^87^ The coders (AG and AQ) were blind (muted videos labeled by LP) to the condition of the videos. Inter-observer reliability was assessed within the two coders for a total of the 25% of the videos and they were in significant agreement (for the four variables, Intraclass Correlation Coefficient (ICC) ≥ 0.91, *p* < 0.00169).

### Quantification and statistical analysis

#### Model 1

A Generalized Linear Mixed Model (GLMM) with number of gazes directed to the speaker as response variables using a Poisson distribution with zero inflation (zero values were present in the response variable) was run using the glmmTMB package.^88^ The subject identity was included as random factor, whereas fixed factors considered were the trial order (sequence in which stimuli were presented), the sex of the tested subject (female/male), the context _receiver_ experienced by the tested subject (social/non-social) and the stimulus context_trigger_ (high-social, low-social and non-social). The trial order was included in both the full and the null model as a control variable. Multicollinearity^88^ was assessed in the GLMMs using the “check_collinearity” function (R package performance 0.4.4). “Low correlation” was present for all fixed factors in the four models (range VIFmin = 1.01; VIFmax = 2.22). The model did not include any interaction for convergence issues.

#### Model 2

A GLMM with number of bouts of self-directed behavior as response variables was run using a Poisson distribution with zero inflated using the glmmTMB package.^88^ The subject identity was included as random factor, whereas the fixed factors considered were the trial order, the sex of the subject, the context_trigger_ and the context_receiver_. The trial order was included in both the full and the null model as a control. Multicollinearity^89^ assessed in the GLMMs using the “check_collinearity” function (R package performance 0.4.4). There was “low correlation” for all fixed factors in the four models (range VIFmin = 1.03; VIFmax = 1.19). The model did not include any interaction for convergence issues.

#### Model 3

A GLMM with presence/absence of yawn responses (in the 3 min after the end of the first call of the stimuli) as response variables was run using a Binomial error distribution. The subject id was included as random factor, whereas fixed factors were the trial order, the context_receiver_ experienced by the subject (social/non-social), the stimulus context_trigger_, and the number of gazes directed to the speaker. The number of gazes served as a check to determine whether the levels of interest toward the stimulus influenced the probability of response. The trial order and the number of gazes directed to the speaker were included in both the full and the null model as a control. Multicollinearity^89^ in the GLMMs was detected using the “check_collinearity” function (R package performance 0.4.4). There was “low correlation” for all fixed factors in the four models (range VIFmin = 1.05; VIFmax = 2.52).

#### Model 4

A GLMM with presence/absence yawn responses (in the 3 min after the end of the first call of the stimuli) was run as response variables using a Binomial distribution. The model included only a subset of entire dataset considering only the playback sessions in which subjects were involved in grooming (social context). Observations considered here only included playback sessions in which the two stimuli presented to the animal were High-social yawns and Grunts. The subject identity was included as random factor, whereas the fixed factors considered were the trial order, the stimulus condition_trigger_ (high-social yawns/grunts), and the sex. The trial order was included in both the full and the null model as a control. There was “low correlation” for all fixed factors in the four models (range VIFmin = 1.01; VIFmax = 1.10).

Models’ significances were tested by comparing the full model to the control model (i.e., only including random and control factor(s)) through the Likelihood Ratio Test (LRT, ANOVA with the “Chisq” argument) and then the *p*-values of each predictor were estimated running LRTs between the full model and the model not containing that predictor.^90^ All statistical comparisons were performed using generalized linear mixed models (GLMM). Significance is indicated in the figures with asterisks (∗*p* < 0.05, ∗∗*p* < 0.01). To assess, models fit and possible overdispersion issues the package DHARMa 0.3.3.073 was used. All pairwise comparisons were performed with the appropriate Bonferroni correction, using the Tukey test.^90^

We initially attempted to include also the duration of the first gaze as the response variable; however, the model did not converge. Therefore, we opted to exclude this variable from the final analysis.

### Additional resources

No additional resources.
