# Supplementary material for: Social engagement modulates wild monkeys’ vocal expressions and the behavioral response to that of others
Source: iScience. 2025 Dec 11;29(1):114408. doi: 10.1016/j.isci.2025.114408 (PMC12800624; doi:10.1016/j.isci.2025.114408)
Supplement: Document S1. Figures S1 and S2, Tables S1–S3 [file mmc1.pdf]

iScience, Volume *29*

Supplemental information

Social engagement modulates wild monkeys' vocal expressions and the behavioral response to that of others

Alice Galotti, Luca Pedruzzi, Martina Francesconi, Alberto Quartesan, Sheleme Abiyou Gamessa, Valentina Serra, Giulio Petroni, Bezawork Afework Bogale, Alban Lemasson, and Elisabetta Palagi

Figure S1 – Picture of the study site, Godot (North-West Shewa zone, Oromia regional state, Ethiopia).

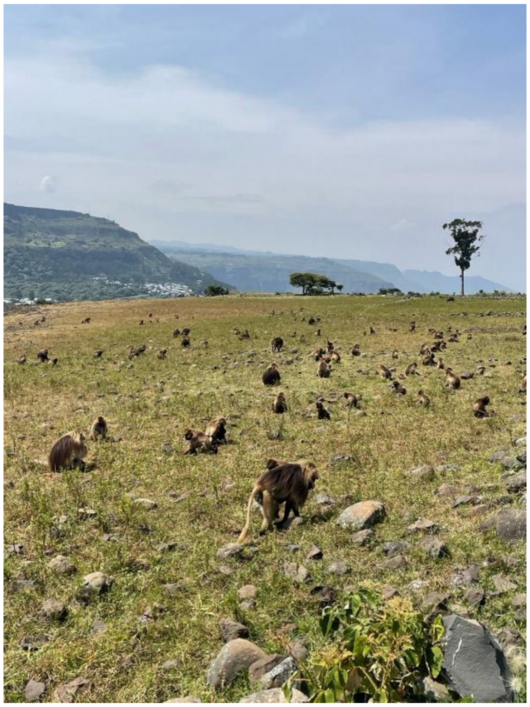

Figure S2 – The graph illustrates how different acoustic parameters contribute to the separation of vocalization categories along the two discriminant functions (LD1 and LD2). The x-axis represents the two discriminant functions (LD1 and LD2), while the y-axis indicates the standardized coefficients of each parameter. The colour gradient, ranging from red to purple, represents the relative weight of each parameter in distinguishing between the vocalization categories, with red indicating the highest contribution and purple the lowest. This visualization helps interpret which acoustic features are most relevant in differentiating high-social, low-social, and non-social yawn vocalizations.

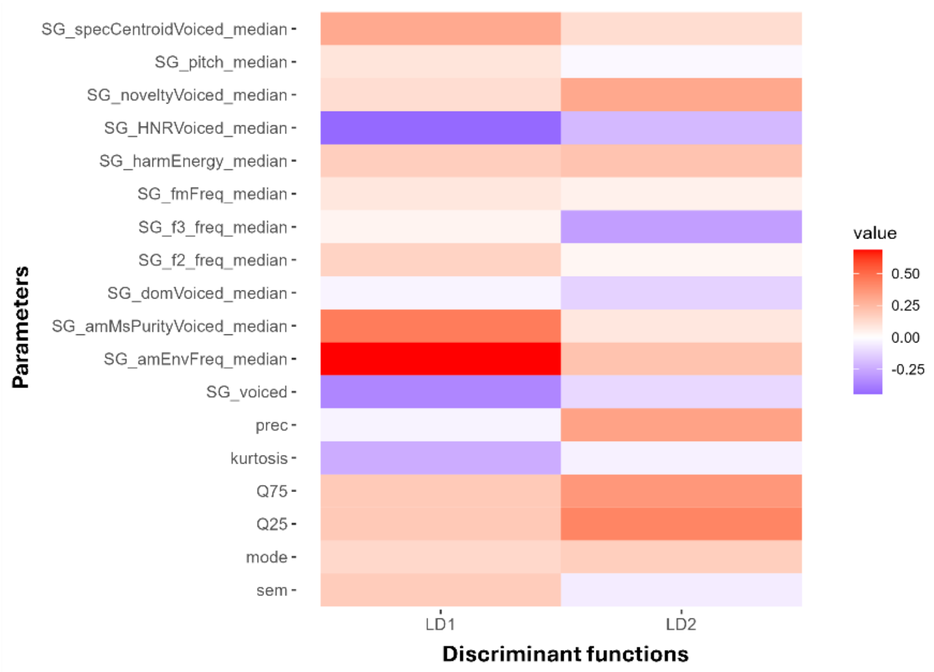

Table S1. Acoustic parameters with PCC ≥ 1 and with normalized distribution that we used to perform discriminant function analysis (DFA). The distribution normality of each of these parameters was confirmed through visual inspection and Shapiro-Wilk tests.

**Parameter name**	**Definition**
**SG_specCentroidVoiced_median**	Median spectral centroid, representing the "center of mass" of the spectrum, indicative of the brightness of the sound.
**SG_pitch_median**	Median fundamental frequency (F0), representing the base frequency of the vocalization.
**SG_noveltyVoiced_median**	Measure of spectral variability over a given time scale, estimated using a self-similarity matrix (SSM).
**SG_HNRVoiced_median**	Median harmonic-to-noise ratio (HNR), indicating the clarity or purity of the voice.
**SG_harmEnergy_median**	Median harmonic energy: the ratio of spectral energy above 1.25 x F0 to the spectral energy below 1.25 x F0 (in dB).
**SG_fmFreq_median**	Median frequency of frequency modulations (FM).
**SG_f3_freq_median**	Median center frequency of the third formant, related to vocal tract resonance.
**SG_f2_freq_median**	Median center frequency of the second formant, related to vocal tract resonance.

**SG_domVoiced_median**	Median dominant frequency, i.e., the frequency with the highest energy.
**SG_amMsPurityVoiced_median**	Purity and frequency of amplitude modulation, estimated via the modulation spectrum.
**SG_amEnvFreq_median**	Frequency (Hz) and depth (0 to 1) of amplitude modulation, estimated from a smoothed amplitude envelope.
**SG_voiced**	Proportion or relative duration of voiced segments within the total signal duration.
**Prec**	Frequency precision of the spectrum.
**Kurtosis**	Measure of spectral kurtosis, indicating the "peakedness" of the spectral distribution.
**Q75**	Third quartile of the spectral distribution (frequency below which 75% of spectral energy is found).
**Q25**	First quartile of the spectral distribution (frequency below which 25% of spectral energy is found).
**Mode**	Mode frequency, i.e., the most frequently occurring frequency.
**Sem**	Standard error of the mean.

Table S2. Descriptive values for the number of looks toward the speaker, number of scratches, and the presence (1) or absence (0) of yawns recorded both before and after the presentation of playbacks including yawning stimuli. Each row represents a playback administration (N = 54). Each subject (sex: 0 = male; 1 = female) was tested under three conditions (H = high arousal; L = low arousal; R = resting) and in two contexts (feeding and grooming). Data are organized by sex (males first, followed by females) and alphabetically within each sex category.

Subject	Sex	Condition	Context	Number of gazes BEFORE	Number of scratches BEFORE	Presence of yawns BEFORE (0/1)	Number of gazes AFTER	Number of scratches AFTER	Presence of yawns AFTER (0/1)
Boss	0	R	feeding	1	0	0	3	0	0
Boss	0	L	feeding	1	0	0	3	0	0
Boss	0	H	feeding	0	0	0	0	0	0
Boss	0	R	grooming	3	1	0	0	0	1
Boss	0	L	grooming	0	0	0	0	1	0
Boss	0	H	grooming	0	0	0	1	1	1

Cry	0	R	feeding	0	0	0	1	0	0
Cry	0	L	feeding	0	0	0	4	0	0
Cry	0	H	feeding	2	0	0	4	1	0
Cry	0	R	grooming	0	1	0	0	0	0
Cry	0	L	grooming	0	0	0	0	0	1
Cry	0	H	grooming	0	0	0	0	1	1
Dark	0	R	feeding	0	0	0	2	1	0
Dark	0	L	feeding	1	1	0	3	0	0
Dark	0	H	feeding	0	0	0	5	0	0
Dark	0	R	grooming	0	0	0	0	0	0
Dark	0	L	grooming	0	0	0	7	0	0
Dark	0	H	grooming	0	0	0	1	2	1
Medium	0	R	feeding	0	0	0	0	0	0
Medium	0	L	feeding	0	0	0	3	0	1
Medium	0	H	feeding	3	0	0	3	0	0
Medium	0	R	grooming	0	0	0	0	0	0
Medium	0	L	grooming	0	0	0	0	0	0
Medium	0	H	grooming	0	0	0	3	0	1
Renato	0	R	feeding	0	0	0	1	0	0
Renato	0	L	feeding	1	0	0	2	0	0
Renato	0	H	feeding	0	0	0	2	0	0
Renato	0	R	grooming	0	1	0	1	0	0
Renato	0	L	grooming	0	0	0	0	0	0
Renato	0	H	grooming	0	2	0	1	2	1
Botola	1	R	feeding	1	0	0	2	0	0
Botola	1	L	feeding	1	1	0	3	2	0
Botola	1	H	feeding	1	1	0	4	1	0
Botola	1	R	grooming	0	0	0	0	0	1
Botola	1	L	grooming	0	0	0	2	0	0
Botola	1	H	grooming	0	0	0	0	1	0
Carla	1	R	feeding	1	0	0	2	1	0
Carla	0	L	feeding	0	0	0	2	1	0
Carla	1	H	feeding	0	0	0	0	3	0
Carla	1	R	grooming	0	0	0	0	1	0
Carla	1	L	grooming	0	1	0	3	2	0
Carla	1	H	grooming	0	1	0	5	0	0
Miriam	1	R	feeding	0	0	0	1	0	0
Miriam	1	L	feeding	0	0	0	1	0	0

Miriam	1	H	feeding	0	0	0	2	0	0
Miriam	0	R	grooming	0	0	0	0	0	0
Miriam	1	L	grooming	0	0	0	3	0	0
Miriam	1	H	grooming	0	0	0	0	0	0
Ravy	1	R	feeding	1	1	0	3	0	0
Ravy	1	L	feeding	0	0	0	1	0	0
Ravy	1	H	feeding	3	1	0	5	0	0
Ravy	1	R	grooming	0	1	0	1	3	0
Ravy	1	L	grooming	0	0	0	1	0	0
Ravy	1	H	grooming	1	1	0	6	1	1

Table S3. Descriptive values for the presence (1) or absence (0) of yawns recorded before and after the presentation of playbacks including grunts (control condition, C) or high-arousal yawning stimuli (high-arousal condition, H), considering only playback sessions conducted in the grooming context. Each row represents a playback administration. For each subject (sex: 0 = male; 1 = female), data are organized by sex (males first, followed by females) and alphabetically within each sex category.

Subject	Sex	Condition	Context	Presence of yawns BEFORE	Presence of yawns AFTER
Boss	0	C	grooming	0	0
Boss	0	H	grooming	0	1
Cry	0	C	grooming	0	0
Cry	0	H	grooming	0	1
Dark	0	C	grooming	0	0
Dark	0	H	grooming	0	0
Medium	0	C	grooming	0	0
Medium	0	H	grooming	0	1
Renato	0	C	grooming	0	1
Renato	0	H	grooming	0	1
Botola	1	C	grooming	0	0
Botola	1	H	grooming	0	0
Carla	1	C	grooming	0	1
Carla	1	H	grooming	0	0
Miriam	1	C	grooming	0	0
Miriam	1	H	grooming	0	0
Ravy	1	C	grooming	0	0
Ravy	1	H	grooming	0	1
